# Oral health and background characteristics in a prison population: the PriOH study

**DOI:** 10.2340/aos.v85.45307

**Published:** 2026-01-16

**Authors:** Kathrine Høyvik Bergum, Emilie Bryne, Linda Maria Stein

**Affiliations:** a Oral Health Centre of Expertise – Rogaland, Stavanger, Norway; b Faculty of Health and Social Science, University of South-Eastern Norway, Borre, Norway; c Department of Clinical Dentistry, UiT The Arctic University of Norway, Tromso, Norway

**Keywords:** Incarceration, social determinants of health, dental anxiety, DMF index, preventive dentistry

## Abstract

**Objectives:**

The Nordic welfare state aims for social security and equality. However, incarcerated people often face vulnerable situations impacting oral health. This study describes oral health and background characteristics of incarcerated people in Norway and explored differences between Nordic and non-Nordic origins.

**Material and methods:**

This cross-sectional study included 326 participants from four prisons in Norway. Dental personnel conducted visual oral examinations and collected questionnaire data on sociodemographic, socioeconomic and health-related background, self-reported oral health and dental anxiety.

**Results:**

The population mainly consisted of young (mean 36.9 ± 11.8) men (95.4%). Almost half had low education (46.4%), reported financial difficulties (37.8%), lacked labor market attachment (45%), and over half (54.8%) experienced physical pain. Dental services were irregularly used (56.2%), and dental anxiety was prominent (39%). Almost half reported poor oral health (46.5%) and clinical measures (mean decayed, missing and filled teeth [DMFT] 11.4 ± 7.4) revealed decayed teeth in four of 10 individuals (39.8%). Group differences showed that non-Nordic individuals had stronger labor market attachment, fewer health challenges, and less caries experience than Nordic individuals.

**Conclusion:**

Diverse socioeconomic backgrounds, untreated caries, poor self-reported oral health, dental anxiety, and irregular dental attendance were common. The group disparities challenge the equity goals of the Nordic Welfare state.

## Introduction

Socioeconomic status is a prominent marker of oral health inequalities [1, 2], and many models on health determinants have been proposed in the literature during the last decades. The World Health Organization (WHO) Social Determinants of Health framework (SDH) aims to understand determinants affecting health and support policymaking by identifying opportunities to address these determinants and reduce health inequities [3–5]. According to the SDH conceptual framework, structural determinants, such as economic, social and welfare policies, can generate social hierarchies and influence the socioeconomic status of individuals within societies [4, 5]. Nordic welfare services are placed at the structural level of determinants, aiming to ensure high social security and equality for all its citizens [6]. Focusing on the Norwegian welfare services, equality is strived for by making education universal without cost, and social security systems exist to support unemployment, pensions, disability needs and necessary healthcare coverage. However, and similar to most countries, oral healthcare is not universal, albeit covered for children and adolescents. Unless specified as a subpopulation with additional needs, adults (above 18 years of age) are expected to pay out of pocket [7].

Although welfare services strive to promote equality, a recent review of the Norwegian population found that social and economic disparities are growing despite the standard of living being high and increasing [8]. This indicates an overall improvement in the population, yet a steep social gradient disproportionately affects those in vulnerable living situations. This social gradient is also visible in the mouth, and oral health is described as a marker revealing one’s social position in society and future health risks [4, 5, 9]. Incarcerated people are often considered a population with vulnerable and challenging living conditions due to limited healthcare access, low-quality care, and unfavorable health outcomes [10–12]. The vast majority of incarcerated people come from challenging and deprived social backgrounds, often with personal histories that can impact their care needs and the treatment required [11]. Reviews have recently found that the oral health of incarcerated people is understudied [10, 13], and insights derived from the general population may not be applicable to the prison population. Such underrepresentation highlights the importance of descriptive epidemiological studies in identifying the distribution of health conditions and disparities within vulnerable settings, such as prisons. In Norway, 40 years have passed since the caries experience of incarcerated people was assessed [14]. Hence, there is a need for updated research on oral health within the prison population [13, 15], enhancing our understanding of determinants of health [4, 5]. The aim of this study was to describe oral health and background characteristics of incarcerated people in Norway, and to explore group differences between Nordic and non-Nordic birth origins.

## Materials and methods

### Study design and participants

This cross-sectional study is part of the PriOH study [16], which had a twofold aim, incorporating both a cross-sectional and an Randomized Controlled Trial (RCT) design. Data for the cross-sectional component were collected from four prisons (three high-security and one low-security – comprising all in the region) in Rogaland County, Norway, from November 2021 to July 2023. The sample was selected using a convenience sampling, where all eligible persons living in the four prisons during the study period were invited to participate, regardless of conviction and length of sentence [16]. The response rate was 41% and the inclusion rate was 29%. The only exclusion criteria were not meeting security standards (in danger of harming themselves or others) and lacking language comprehension (unable to verbally understand Norwegian or English).

### Data collection and variables

Trained dental personnel (1 dentist, 6 dental hygienists and 3 dental assistants) collected all data in a prison visiting room. All dental personnel were calibrated by critically assessing the study protocol and questionnaire, discussing and practicing clinical examination procedures, which included mean number of decayed, missing and filled teeth (DMFT) measurements [16, 17]. For DMFT data collection procedures, training involved collectively reviewing the standardized measurement criteria [17], completing a practical case exercise, and group discussions to ensure agreement consistency in documentation.

Firstly, participants’ oral health was assessed by dental personnel (dentists or dental hygienists) using a dental mirror, a blunt probe, cotton rolls, and light appropriate to dental loupes but attached to regular glasses. This clinical oral health assessment included the number of DMFT, excluding third molars [17]. Decayed teeth (DT) were defined as clinical findings consistent with caries grade 3–5, secondary caries, root caries and root remnants (a tooth with non-functional occlusal or incisal surface). Regardless of the reason, all missing teeth (MT) were registered as missing. FT included all filling materials, crowns, and fixed dental prosthesis abutments. FT with caries or secondary caries were defined as DT.

After the oral health assessment, a comprehensive questionnaire was verbally administered in either Norwegian or English. The questionnaire included background characteristics on sociodemographic, socioeconomic and health-related questions, as well as self-reported oral health on a 5-point Likert scale (trichotomized into good, fair, and poor) and dental anxiety. Dental anxiety was assessed through the modified dental anxiety scale (MDAS) [18, 19], consisting of five multiple-choice questions with a response ranging from 1 (not anxious) to 5 (extremely anxious), with a total score ranging from 5 to 25. The scores are categorized as not anxious (< 10), moderately anxious (10–18), and severely anxious (≥ 19) following cut-off scores applied in a different prison [20] and Norwegian study [21].

### Ethics

The study was approved by the Norwegian Agency for Shared Services in Education and Research (ID: 300281), the Regional Committee for Medical and Health Research Ethics in Western Norway (REK no: 282231), and the Norwegian Correctional Service, Norway. All participants received verbal and written information about the study, and the dental personnel repeated the information to ensure informed consent. Due to the likelihood of low literacy levels in prison populations [22], data are often collected through structured interviews [13]. To ensure literacy levels did not compromise this data collection, dental personnel followed procedures and read all questionnaires aloud, subsequently noting participants’ answers.

### Statistics

All statistical analyses were performed using the IBM SPSS Statistics software version 29.0 (Armonk, NY: IBM Corp). Descriptive statistics (frequencies and means with corresponding standard deviations) were calculated for all participants and separately for the Nordic and non-Nordic groups to highlight differences in having had access to a Nordic welfare system. Cross tabulation was used to explore the group differences between Nordic and non-Nordic. The statistical significance of differences was determined using the Chi-square test, the Mann–Whitney U test or the Fisher’s exact test. For cases with large standard deviations, minimum and maximum values were included. The reliability of MDAS was indicated by a Cronbach’s alpha of 0.91 (*n* = 310) and a mean inter-item correlation of 0.68.

## Results

### Sociodemographic and socioeconomic background

The prison population’s sociodemographic background is presented in Table 1. Of the 326 participants, most were male (95.4%), age ranging from 18 to 67 years (mean 36.9 ± 11.8). Most participants (86.8%) received the questionnaire in Norwegian. The majority of the sample (67.2%) were born in Nordic countries (not tabulated). The non-Nordic population originated from different regions, primarily representing Eastern Europe (*n* = 43), the Middle East (*n* = 21) and Africa (*n* = 18) (not tabulated). In the non-Nordic population, almost two-thirds (63.3%) had immigrated to Norway after the age of 19 (not tabulated). Half (51.3%) had served a prison sentence before. Roughly one fourth (23.5%) were on remand, and three fourths (76.2%) had started serving their prison sentence. Most respondents (70.2%) lived in a high-security prison.

**Table 1:** Sociodemographic background.

	Overall		Nordic		Non-Nordic		*p values*
	*N*	%	*N*	%	*N*	%	
**Age**	326	36.9 (11.8)^1^	219	36.7 (11.8)^1^	107	37.3 (11.8)^1^	0.66^3^
**Gender** ^a^							0.007* ^4^
Male	311	95.4	204	93.2	107	100	
Female	14	4.3	14	6.4			
Other	1	0.3	1	0.5			
**Partner status**							0.50^4^
Partner	117	36.1	76	34.9	41	38.7	
No Partner	207	63.9	142	65.1	65	61.3	
**Self-owned house** ^b^							0.20^4^
Yes	74	32.9	52	35.9	22	27.5	
No	151	67.1	93	64.1	58	72.5	
**Household composition** ^c^							1.00^4^
Lived with someone	187	58.4	128	58.4	59	58.4	
Lived alone	133	41.6	91	41.6	42	41.6	
**Children**							0.47^4^
Yes	181	56	188	54.6	63	58.9	
No	142	44	98	45.4	44	41.1	
**Children under 18**							0.004* ^5^
Yes	145	81.5	89	75.4	56	93.3	
No	33	18.5	29	24.4	4	6.7	
**Lived with during childhood** ^d^							
**Mother and father**							< 0.001** ^4^
Yes	172	54.6	105	48.4	67	68.4	
No	143	45.4	112	51.6	31	31.6	
**One parent**							< 0.001** ^**4**^
Yes	188	37.5	95	43.8	23	23.5	
No	197	62.5	122	56.2	75	76.5	
**Other** ^e^							0.002* ^4^
Yes	81	25.6	45	20.6	36	36.7	
No	235	74.4	173	79.4	62	63.3	
**Number of times living in prison**							0.06^4^
= 1	154	48.7	99	45.2	55	56.7	
> 1	162	51.3	120	54.8	42	43.3	
**Imprisonment**							0.49^4^
Remand	74	23.5	49	22.5	25	25.8	
Sentence	240	76.2	169	77.5	71	73.2	
Preventive detention	1	0.3			1	1.0	
**Prison security**							0.002* ^**4**^
Low	97	29.8	77	35.2	20	18.7	
High	229	70.2	142	64.8	87	81.3	
**Length of sentence** (months)	236	26.6 (31.0)^1^ (1.0–168.0)^2^	166	22.2 (26.5)^1^ (1.0–144.0)^2^	70	37.1 (38.0)^1^ (1.25–168.0)^2^	0.004* ^**3**^
**Sentence already served** (months)	309	7.7 (14.2)^1^ (0.25–122.0)^2^	217	5.3 (10.2)^1^ (0.25–78.0)^2^	92	13.4 (19.7)^1^ (0.25–122.0)^2^	< 0.001** ^**3**^

a Only male and female included in the Pearson’s Chi-Square test;

b Privately rented home, publicly rented home, temporary home, on the street, at friends’ or family members’ house, other;

c Before prison;

d Who did you mainly live with until you were 18 years old?

e Adoptive parents, foster parents, grandparents, other relatives, institution, other.

1 Mean (SD);

2 (Minimum-Maximum);

3 Mann-Whitney Test;

4 Pearson’s Chi-Square test;

5 Fisher’s Exact Test.

* *P* < 0.05;

** *P* < 0.001.

Bold values indicate statistical significance at either p < 0.05 or p < 0.001

The socioeconomic background is shown in Table 2. Almost half of the participants (46.4%) had not completed high school, were unemployed for the last 12 months prior to imprisonment (45.3%), and had an annual income under 300.000 NOK (49.8%). Roughly one in three (37.8 %) found it very hard or hard to make ends meet with their annual income before incarceration, and the greater half (53.8%) reported welfare support as an essential source of income.

**Table 2:** Socioeconomic background.

	Overall		Nordic		Non-Nordic		*p values*
	*N*	%	*N*	%	*N*	%	
**Education** ^a^							0.40^1^
College or more	52	16.2	34	15.6	18	17.5	
High school	120	37.4	87	39.9	33	32.0	
Elementary school or less	149	46.4	97	44.5	52	50.5	
**Employment** ^b^							< 0.001** ^1^
Fulltime	132	41.8	77	35.3	55	56.1	
Parttime	41	13.0	26	11.9	15	15.3	
Unemployed	143	45.3	115	52.8	28	28.6	
**Annual income NOK** ^b^							0.01* ^1^
0–49.000	21	7.4	12	5.9	9	11.2	
50.000–99.000	30	10.5	14	6.8	16	20.0	
1000.000–199.000	39	13.7	31	15.1	8	10.0	
200.000–299.000	52	18.2	43	21.0	9	11.2	
300.000–399.000	51	17.9	39	19.0	12	15.0	
400.000–499.000	29	10.2	20	9.8	9	11.2	
500.000 or more	63	22.1	46	22.4	17	21.4	
**Income adequacy** ^b^							0.50^1^
Easy	122	39.7	82	38.0	40	44.0	
Fair	69	22.5	48	22.2	21	23.1	
Hard	116	37.8	86	39.8	30	33.0	
**Income sources** ^b^							
**Labor income**							< 0.001** ^1^
Yes	149	47.5	86	39.4	63	65.6	
No	165	52.5	132	60.6	33	34.4	
**Welfare support** ^c^							< 0.001** ^1^
Yes	169	53.8	140	62.4	29	30.2	
No	145	46.2	78	35.8	67	69.8	
**Other support/sources** ^d^							0.69^1^
Yes	39	12.4	26	11.9	13	13.5	
No	275	87.6	192	88.1	83	86.5	
**Childhood economic perception**							0.09^1^
Wealthy	27	8.9	22	10.3	5	5.4	
Stable	195	63.9	140	65.7	55	59.8	
Poor	83	27.2	51	23.9	32	34.8	
**Work in prison**							0.89^1^
Yes	205	65.1	142	64.8	63	65.6	
No	110	34.9	77	35.2	33	34.4	
**Education in prison**							< 0.001** ^1^
Yes	118	37.7	64	29.4	54	56.8	
No	195	62.3	154	70.6	41	43.2	

a Highest completed education;

b Before prison;

c Unemployment benefits, sickness benefits, disability benefits, pension, social welfare support, scholarship/study loan/legacy;

d Supported by others, used savings, other support/grants.

1 Pearson’s Chi-Square test.

* *P* < 0.05;

** *P* < 0.001.

Bold values indicate statistical significance at either p < 0.05 or p < 0.001

### Health-related background

The health-related background characteristics of the prison population is described in Table 3. Just over two-fifths (43.7%) reported physical illness. Over half (54.8%) reported that the physical pain lasted for more than 6 months, whereas about half of these (55.0%) reported that pain largely affected them. Prescriptive medication was used by half of the participants (55.2%). Slightly over half (52.6%) used illicit substances outside of prison.

**Table 3:** Health-related background.

	Overall		Nordic		Non-Nordic		*p values*
	*N*	%	*N*	%	*N*	%	
**Physical illness**							0.66^1^
Yes	135	43.7	97	44.5	38	41.8	
No	174	56.3	121	55.5	53	58.2	
**Physical pain** ^a^							0.32^1^
Yes	171	54.8	124	56.6	47	50.5	
No	141	45.2	95	43.4	46	49.5	
**Extent of physical pain impact**							0.18^1^
Little extent	34	19.9	28	22.6	6	12.8	
Some extent	43	25.1	33	26.6	10	21.3	
Great extent	94	55.0	63	50.8	31	66.0	
**Prescriptive medication**							< 0.001** ^1^
Yes	174	55.2	138	63.0	36	37.5	
No	141	44.8	81	37.0	60	62.5	
**Illicit substance use** ^b c^							0.05* ^1^
Yes	163	52.6	121	56.3	42	44.2	
Never	147	47.4	94	43.7	53	55.8	
**Previous Treatment/care** ^d^							
**Substance treatment**							< 0.001** ^1^
Yes	80	26.1	75	34.6	5	5.6	
No	227	73.9	142	65.4	85	94.4	
**Drug-assisted rehabilitation**							0.01*^1^
Yes	27	8.8	25	11.5	2	2.2	
No	280	91.2	192	88.5	88	97.8	
**Mental healthcare**							<0.001** ^1^
Yes	95	30.9	81	37.3	14	15.6	
No	212	69.1	136	62.7	76	84.4	
**Physical/Somatic treatment**							0.36^1^
Yes	39	12.7	30	13.8	9	10.0	
No	268	87.3	187	86.2	81	90.0	
**General practitioner** ^e^							0.01* ^1^
Yes	274	87.8	199	90.9	75	80.6	
No	38	12.2	20	9.1	18	19.4	
**Dental health service utilization** ^e^							0.33^1^
Regular	138	43.8	92	42.0	46	47.9	
Irregular	177	56.2	127	58.0	50	52.1	
**Dental health service utilization during imprisonment**							0.19^1^
Yes	111	35.2	72	32.9	39	40.6	
No	204	64.8	147	67.1	57	59.4	

a For more than 6 months;

b Before prison;

c Illicit substance us include drugs and misuse of prescription medication (i.e. taking beyond prescribed limits, using for non-medical reasons, or obtaining without prescription);

d After turning 18 years old;

e Outside prison.

1 Pearson’s Chi-Square test.

* *P* < 0.05;

** *P* < 0.001.

Bold values indicate statistical significance at either p < 0.05 or p < 0.001

More than half (54.4%) had received healthcare after the age of 18 and before incarceration (not tabulated), with the largest share of health services within mental healthcare (30.9%) and substance treatment (26.1%). About two-thirds (64.8%) had not attended the prison dental health service. More than half (56.2%) did not use dental health services regularly outside the prison context. The largest share of reasons was attributed to dental anxiety (27.1%), no perceived need (22.4%) and inability to afford it (21.2%) (Appendix 1). One-fourth (24.7%) reported other reasons for not utilizing dental health services.

### Oral health

Oral health variables are shown in Table 4. The mean DMFT was 11.4 (±7.4). Close to two out of five (39.8%) had untreated decay. FT was the dominant component of the DMFT index (7.3 ± 5.3). Almost half (46.5%) of the population regarded self-reported oral health as poor. Just under one-third (29.4%) experienced moderate dental anxiety, and one-tenth (10%) severe dental anxiety.

**Table 4:** Oral health.

	Overall						Nordic						non-Nordic						*p values*
	*n*	%	Mean	SD	Min	Max	*n*	%	Mean	SD	Min	Max	*n*	%	Mean	SD	Min	Max	
**Number of teeth**	324		25.4	4.1	5	28	218		25.3	4.2	5	28	106		25.5	4.0	5	28	0.46^1^
**DMFT**	324		11.4	7.4	0	28	218		12.4	7.5	0	28	106		9.4	6.8	0	28	< 0.001** ^1^
**DT**	324		1.4	3.2	0	28	218		1.5	2.9	0	15	106		1.2	2.9	0	20	0.09^1^
**MT**	324		2.7	4.1	0	28	218		2.9	4.5	0	28	106		2.4	3.7	0	15	0.36^1^
**FT**	324		7.3	5.3	0	23	218		8.0	5.4	0	23	106		5.8	4.6	0	22	< 0.001** ^1^
**DT ≥ 1**																			0.13^2^
**DT = 0**	195	60.2					125	57.3					70	66.0					
**DT = ≥ 1**	129	39.8					93	42.7					36	34.0					
**Self-reported oral health**																			0.87^2^
Good	97	30.7					66	30.4					31	31.3					
Fair	72	22.8					48	22.1					24	24.2					
Poor	147	46.5					103	47.5					44	44.5					
**Dental anxiety** ^a^																			
Total mean score	310		9.6	5.5			215		10.0	5.6			95		8.8	5.0			0.08^2^
Not anxious	188	60.6					122	56.7					66	69.5					
Moderately anxious	91	29.4					71	33.0					20	21.0					
Severely anxious	31	10.0					22	10.3					9	9.5					

a MDAS score: < 10 = not anxious, 10–18 = moderately anxious and ≥ 19 = severely anxious.

1 Mann-Whitney Test.

2 Pearson’s Chi-Square test.

*P < 0.05.

** *P* < 0.001.

Bold values indicate statistical significance at either p < 0.05 or p < 0.001

### Group differences

All tables depict group differences between Nordic and non-Nordic incarcerated people. More of the non-Nordic participants were in high security prisons, and those who had a sentence had served longer time on average in prison at the point of the data collection than the Nordic group (Table 1). Non-Nordic individuals were more likely to have participated in prison educational programs, been employed 12 months prior to incarceration, and reported labor as their essential income source before imprisonment (Table 2). Subsequently, Nordic individuals were more likely to rely on welfare support as an essential income source before imprisonment (Table 2). The Nordic group was more likely to use prescriptive medication, having used illicit substances before imprisonment, having received substance treatment, drug-assisted rehabilitation and mental healthcare and having a general practitioner (Table 3). Lastly, significant group differences in the mean DMFT score between Nordic and non-Nordic were found, with more people of Nordic origin scoring higher on DMFT and FT scores than the non-Nordic participants (Table 4).

## Discussion

This is the first study examining the DMFT score in a Norwegian prison population in 40 years [14]. The findings update knowledge of oral health in a subpopulation often not included in larger oral health studies [23, 24] – a key novel feature of this work. The findings indicate an improvement of clinically measured oral health since 1984 [14], but challenges with dental service attendance and dental anxiety still seem to persist in the prison population in Norway. Moreover, this study also included participants’ self-reported oral health measures, adding a subjective dimension to this population’s relationship to oral health.

Not surprisingly, the mean number of DT and MT in the Norwegian prison population had decreased among incarcerated people since the study 40 years ago [14]. However, the observed caries experience within the current population indicates a considerable variation, with some people experiencing a significantly higher burden of caries than others. Understanding our findings in the light of previous prison oral health research is challenging due to the limited numbers. Available recent prison population studies from Portugal [25], Finland [26], and Russia [27] report a higher mean DMFT score than in Sweden [28], Scotland [29], Kosovo [30] and this study. The variation in the mean DMFT score reported across these countries could be attributed to varying accessibilities to dental health services and different treatment strategies. The incarcerated people’s diverse background and, consequently, their different determinants of oral health, could also account for some of the differences.

Surprisingly, in this study, FT constituted the majority of the DMFT score, particularly in the Nordic population, which contrasts with a review noting DT and MT as dominant components [13]. The higher proportion of treated teeth in this study could be attributed to having had access to, and having used, dental health services. Nevertheless, around two out of five in this prison population had untreated caries, which indicates a significant need for dental treatment. Although many in this study population needed dental treatment, most participants had not used dental health services while imprisoned, in spite of the fact that examination and necessary treatment were free of cost through the Norwegian Dental Care Act when imprisonment exceeds 3 months [7]. Thus, the study’s findings also shed light on access to dental services during incarceration, suggesting that simply removing financial barriers may not be sufficient for these individuals to achieve and maintain good oral health. Moreso, treating dental caries by filling teeth alone is not a sustainable solution. Good oral health is achieved by both disease preventive and health promotional measures ingrained in daily oral health routines. Given the prison’s focus on reintegration and routines, the prison arena could facilitate collaboration across services and promote good oral health routines by providing oral hygiene products, giving incarcerated people personalized oral health education, and ensuring accessible dental services. However, more research on prison dental service accessibility is warranted to explore this knowledge gap further.

Through self-reported measures, this study identified that almost half of the prison population reported their oral health as poor. Attending to incarcerated people’s oral health is of concern, considering this percentage is notably higher than Norwegian population studies reporting a range from 7 to 13% [31, 32]. The discrepancies in self-reported poor oral health between incarcerated and non-incarcerated individuals could indicate a specific challenge for the prison population. Such a claim is further supported by other studies with prison populations in Australia and Sweeden, where 27% and 73% reported their oral health as poor [28, 33]. One’s understanding of what constitutes good and poor oral health can differ across people as subjective experiences, psychological factors, culture and personal expectations influence self-reported oral health. In a Swedish study, the primary reason (55%) for dissatisfaction with oral health among incarcerated individuals was aesthetic and functional factors, such as tooth color, alignment, and the number of teeth [28]. In contrast, an Australian study revealed that many incarcerated individuals overestimated their oral health compared to clinical findings [33]. Qualitative research could provide deeper insights into the oral health needs of this population that may not be fully capture by clinical measurs.

Experiencing poor oral health can be a significant burden in everyday life, affecting both physical and psychological health and social well-being. Moreover, the associated stigma can be particularly problematic considering this young population (mean age of 37). Furthermore, MT and poor oral health can be barriers when applying for jobs and rental apartments, which are essential for reintegration into society [34].

Dental anxiety was prominent in this population, which is in line with findings from the Norwegian prison study from 1984 [14]. In a national survey of dental health services in Norwegian prisons, dental anxiety was considered as the most common barrier for dental attendance in the prison context, by prison dentists and prison staff [35]. By combining moderate and severe cut-off scores, results revealed that dental anxiety affected almost 40% of this study’s population. Previous studies assessing dental anxiety in prison populations have similarly found high rates [20, 36]. Within the 40% of dental anxiety of this study population, 10% reported severe dental anxiety, which is considerably higher than the 2.9% identified in the general Norwegian population [21]. Yet, background characteristics such as irregular use of dental health services and low income were more typical for those with severe dental anxiety [21] and resonate with the descriptive findings in this prison population.

The background characteristics display the diversity of this population. Still, socioeconomic challenges, such as low education levels, unemployment, and financial difficulties, were common. The socioeconomic challenges in this population align with previous studies from several countries, which have also found low education levels [25, 26, 37] and high unemployment rates before incarceration [28, 29, 38]. Moreover, a Norwegian population study found that lower education or income levels were associated with poorer oral and general health [23]. Although general health was not directly measured within this study population, physical pain affected more than half of the population. Forty per cent reporting physical pain may seem high, Norwegian and Nordic population studies show similar results [39–41].

This study population’s reported use of health services varied, yet it depicted an irregular use of dental health services. People in Norway have more extensive healthcare rights than in non-Nordic countries, which is attributed to the Nordic welfare system acting as a structural determinant. However, unlike general healthcare, dental health services differ as they are not subsidized for most adults. Over half of the participants attended dental health services irregularly before incarceration. Although this finding indicates an increase in dental attendance since 1984, where 80% attended dental health services irregularly [14], incarcerated people are still an underserved population within the dental services. Interestingly, approximately one-fourth reported ‘other reasons’ for not using dental health services in this study. Even though this question is extracted from a Norwegian population study, given the high rates responding ‘other reasons’, we argue this question does not seem to fully capture the irregular dental attendance specific to this prison population. A qualitative study [42] found that stigma and inconvenient opening hours were obstacles to accessing dental health services outside prison. Addressing our findings at a population level, and within the political and structural landscape of welfare services, prioritizing people in the most vulnerable living conditions may help to close the gap in disparities and support oral health equity.

The group differences within the prison population presented more non-Nordic individuals with an attachment to the labor market. Regarding health issues, the non-Nordic group alluded to better health as they reported less experience with healthcare systems, less use of substances, and had a lower mean DMFT score than the Nordic individuals. Considering differences in the financial situation and DMFT score between the Nordic and non-Nordic populations, one might have expected a difference in dental health service utilization. Still, both populations attended dental health services irregularly. This suggests that determinants beyond financial constraints play a role in dental health service utilization, as addressed by the SDH framework.

### Strengths and limitations

This study has some limitations. Firstly, the study design within a prison context has some shortcomings due to the lack of dental equipment and high security measures. Dental radiographic images were not included in the examination, which could potentially affect the validity of the results. Nevertheless, it is likely to assume that more caries would have been detected with radiographic images, as absence of radiographs has a 44% probability that the caries decay value will be lower than the actual value [43]. We acknowledge that oral health encompasses more than the DMFT score, which was the only clinical measure in this study, and we attempted to mitigate our clinical measurement shortcomings by including self-reported subjective oral heath scores. However, the reader should note that participants completed the oral examination before conducting the questionnaire, which may have influenced participants’ self-reported oral health. As participants were recruited for an RCT, selection bias might have been present, limiting the generalizability to broader prison populations. Also, the exclusion criteria of language and safety measures could affect generalizability. Lastly, this study’s descriptive analysis does not permit conclusions about causality or statistical associations. However, the findings highlight the need for future studies to investigate statistical associations, causal relationships and underlying mechanisms affecting incarcerated people’s oral health.

The study’s strength is the collection of questionnaire data through standardized interviews, which facilitated literacy difficulties and likely minimizing missing data. The standardized interview also allowed dental personnel to explain questions and ensure participants’ ability to withdraw from answering uncomfortable questions. Nevertheless, verbal responses might have influenced participants’ comfort level and affected reliability. Another strength is that, despite severe and moderate levels of dental anxiety, a substantial number of participants consented to participation.

## Conclusion

Oral health challenges such as untreated caries, poor self-reported oral health, dental anxiety, and irregular use of dental health services were common. Moreover, the Nordic population had higher caries experience, reported more health challenges, and were less attached to the labor market than the non-Nordic individuals. Although this prison population had diverse backgrounds, a large proportion of these reported a socioeconomic position that is considered low within a welfare state. In this sample, the observed group differences suggest a paradoxical outcome of the Nordic welfare state: despite its aim to reduce social and health inequalities, it appears to have fallen short within this prison population.

Future research should look deeper into tailored preventive and promotive initiatives to enhance oral health and explore existing barriers to dental attendance in prisons.

## Supplementary Material


