# Supplementary material for: Oral health and background characteristics in a prison population: the PriOH study
Source: Acta Odontol Scand. 2026 Jan 16;85:45307. doi: 10.2340/aos.v85.45307 (PMC12821324; doi:10.2340/aos.v85.45307)
Supplement: Supplementary file 1 [file AOS-85-45307-s1.pdf]

Supplementary material has been published as submitted.
It has not been copyedited or typeset by Acta
Odontologica Scandinavica.

Appendix A. Reasons for not using dental health service regularly

	Overall		Nordic		non-Nordic		
	N	%	N	%	N	%	*p*
**Reasons for not regularly using the dental health service** [a]							
**Not called in**							0.72[1]
Yes	10	11.8	8	13.1	2	8.3	
No	75	88.2	53	86.9	22	91.7	
**No need**							0.04*[2]
Yes	19	22.4	10	16.4	9	37.5	
No	66	77.6	51	83.6	15	62.5	
**Long waitlist**							0.02*[1]
Yes	5	5.9	1	1.6	4	16.7	
No	80	94.1	60	98.4	20	83.3	
**Travel issues**							0.49[1]
Yes	2	2.4	1	1.6	1	4.2	
No	83	97.6	60	98.4	23	95.8	
**Not had time**							1.0[1]
Yes	10	11.8	7	11.5	3	12.5	
No	75	88.2	14	23.0	21	87.5	
**Can not afford it**							0.77[1]
Yes	18	21.2	14	23.0	4	16.7	
No	67	78.8	47	77.0	20	83.3	
**Afraid**							0.28[1]
Yes	23	27.1	19	31.1	4	16.7	
No	62	72.9	42	68.9	20	83.3	
**Health problems**							0.57[1]
Yes	4	4.7	4	6.6	0	0	
No	81	95.3	57	93.4	24	100	
**Other reasons**							0.78[1]
Yes	21	24.7	16	26.2	5	20.8	
No	85	75.3	45	73.8	19	79.2	

[a]: outside prison [1]: Fisher Exact test [2]:Person Chi-Square test *: $P < 0.05$ **: $P < 0.001$
